# Concomitant Left Ventricular and Popliteal Thrombectomy With Coronary Artery Bypass Grafting

**DOI:** 10.1177/23247096261491190

**Published:** 2026-09-16

**Authors:** Jeffrey Balian, Dominick D’Antuono, John Conrad, Ali Gheissari, Vahram Ornekian

**Affiliations:** 1 Center for Advanced Surgical and Interventional Technology, Department of Surgery, 8783 University of California, Los Angeles, CA, United States of America; 2 Division of Cardiothoracic Surgery, Department of Surgery, Adventist Health Glendale, Los Angeles, CA, United States of America; 3 Division of Vascular Surgery, Department of Surgery, 24518 Providence Saint Joseph Medical Center, Los Angeles, CA, United States of America

**Keywords:** left ventricular thrombus, coronary bypass, popliteal thrombectomy, left ventriculotomy

## Abstract

Left ventricular (LV) thrombus in patients with severe coronary artery disease (CAD) poses a difficult decision regarding the safety and timing of revascularization. We report a 61-year-old woman presenting with non-traumatic right ankle pain, a consequence of popliteal artery cardioembolism. Laboratory findings included elevated troponin (549 ng/L), white blood cell count (11.9 k/μL), and hemoglobin A1C (11.5%). Echocardiography revealed distal anteroseptal and apical akinesis with associated LV thrombus, and cardiac catheterization demonstrated underlying multivessel CAD. Given the embolic risk and acute limb malperfusion, LV thrombectomy via apical ventriculotomy, three-vessel coronary bypass, and right popliteal artery thrombectomy with vein patch angioplasty in a single operative setting. The patient recovered without complication and was discharged to a rehabilitation facility on postoperative day six. At three-month follow-up, she remained free of recurrent thromboembolism with normalized LV systolic function and complete resolution of lower extremity symptoms. This case demonstrates the safety of concomitant surgical management of LV thrombus, coronary revascularization, and peripheral thrombectomy in appropriately selected patients, addressing both cardiac and limb ischemia while minimizing embolic risk.

## Introduction

Despite improvements in reperfusion therapy, left ventricular (LV) thrombus formation is a dreaded complication following anteroapical myocardial infarction, occurring in an estimated 4 to 39% of cases.^1^ Furthermore, depending on morphology and mobility, LV thrombus development has been associated with a 19% risk of all-cause mortality and a 37% risk of major adverse cardiovascular events, necessitating prompt intervention for these complex patients.^2^ Current guidelines recommend the initiation of therapeutic anticoagulation for three months, followed by subsequent imaging.^3,4^ However, for mobile LV thrombi with concurrent coronary artery disease (CAD), urgent operative management may be suggested due to increased risks of morbidity and embolization.^1,4-6^ Such patients may also demonstrate thromboembolic burden peripherally, which may prompt embolectomy for limb salvage.^7^ We report a favorable outcome following concomitant LV ventriculotomy, popliteal artery thrombectomy, and three-vessel coronary artery bypass grafting (CABG). This case can provide cardiac and vascular teams with valuable insight for managing post-infarction sequelae in patients with multivessel CAD and LV thrombi.

## Case Presentation

A 61-year-old female patient with no known past medical history presented to our emergency department with a 10-day history of non-traumatic right ankle pain. She noted new-onset discoloration of the right great toe with associated acute pain, which appeared pale and cyanotic upon examination. She denied similar episodes in the past and denied trauma to the affected extremity. The patient was free of edema, chest pain, palpitations, and shortness of breath, and denied a history of such symptoms.

She has had previous ovarian cyst surgery; otherwise, she has not had any significant surgical procedures. She reported no other significant medical history.

Duplex ultrasonography performed in the emergency department revealed concern for a thrombus within the popliteal artery and a lack of blood flow in the right infrapopliteal vasculature. The patient was systemically heparinized, and a CT angiogram of the right lower extremity was performed, demonstrating occlusion of the right popliteal artery, distal reconstitution of flow, and diffuse anterior popliteal artery disease (Figure 1). Laboratory results were significant for a white blood cell count (11.9 k/μL), troponin I (549 ng/L), and hemoglobin A1C (11.5%).

**Figure 1.**
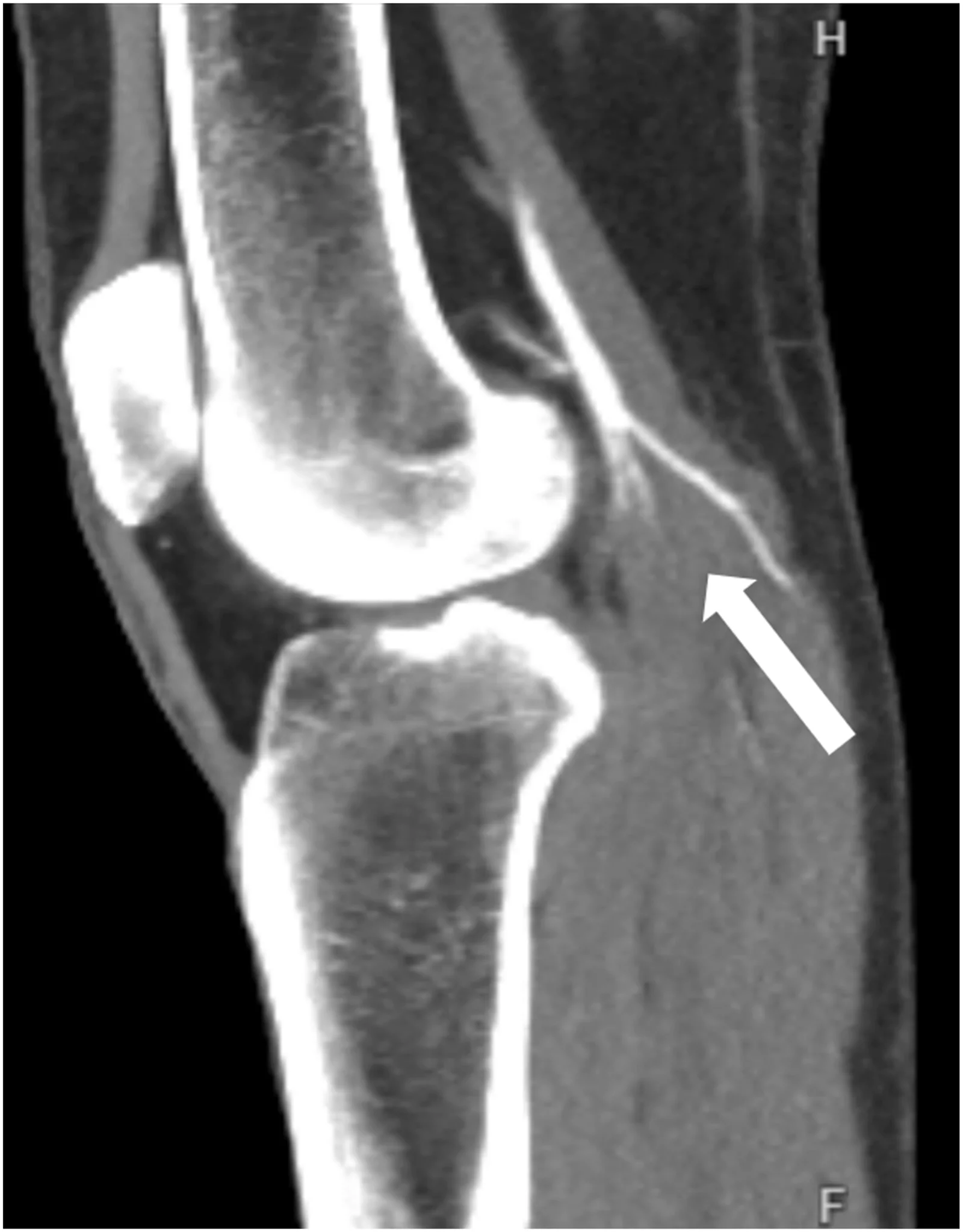
Computed tomography angiogram of the right leg. The white arrow demonstrates occlusion of the right popliteal artery

Further evaluation identified newly diagnosed, poorly controlled diabetes mellitus, as evidenced by a markedly elevated hemoglobin A1C, which may have contributed to the patient’s extensive coronary and peripheral vascular disease. A hypercoagulability workup was also initiated to evaluate for an underlying prothrombotic disorder. Serial troponin measurements were obtained as part of her cardiac evaluation, and the elevated troponin level was consistent with a non-ST-elevation myocardial infarction (NSTEMI), prompting further coronary evaluation.

Given the acute presentation of elevated troponin and limb malperfusion, an emergent ischemic workup was undertaken. The transthoracic echocardiography performed in the emergency room demonstrated normal LV size and wall thickness and mildly reduced LV systolic function. Two highly mobile, pedunculated LV thrombi were noted in the apex, measuring 1.4 x 1.0 cm and 1.0 x 0.8 cm in size (Figure 2, Supplemental Figure 1). Ejection fraction was estimated between 45 and 50%. The distal anteroseptal wall and apex were akinetic, without any other defects in wall motion. Right ventricular size and systolic function were normal. Mild mitral and tricuspid regurgitation was noted. No pericardial effusion was present.

**Figure 2.**
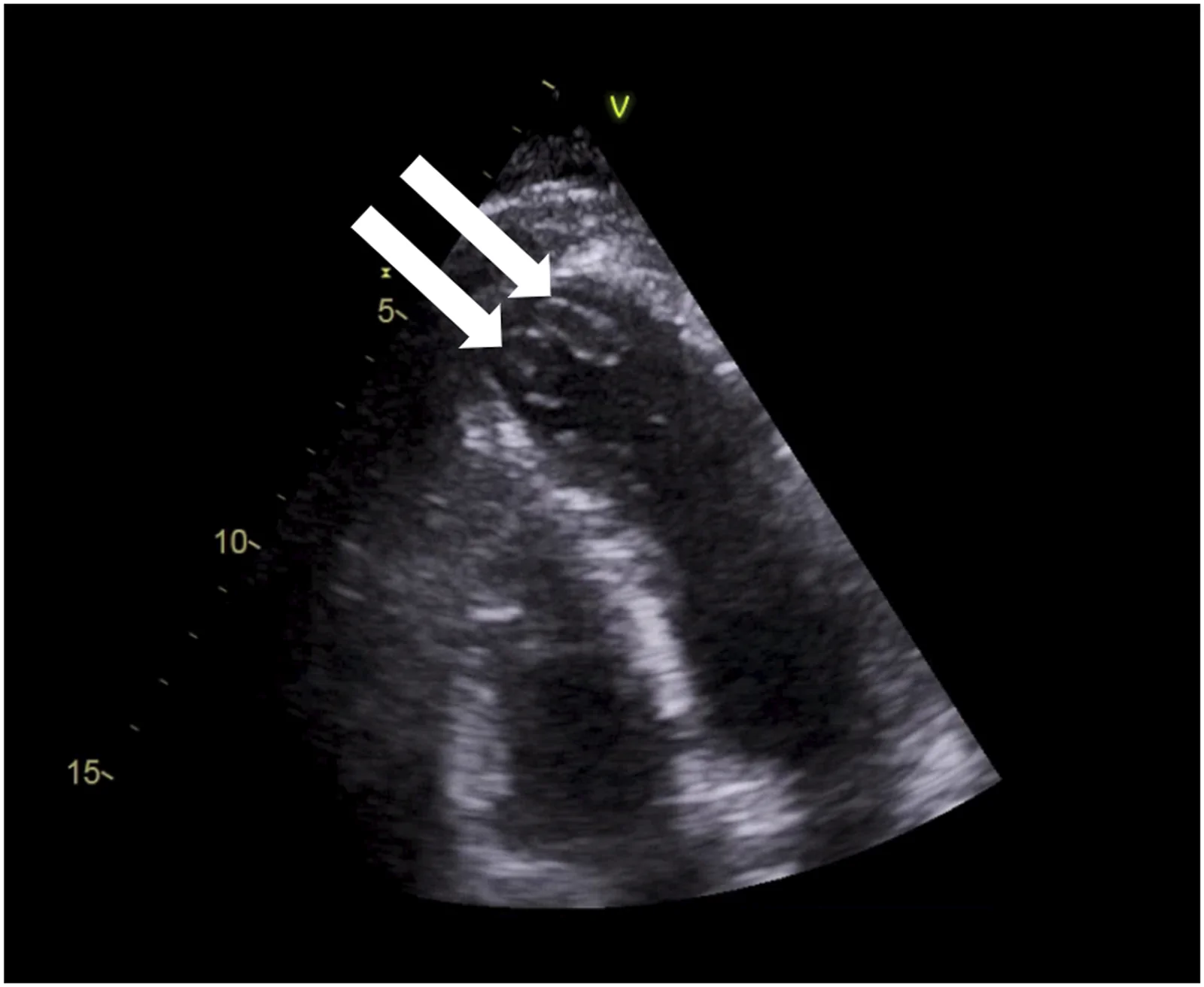
Four-chamber view of transthoracic echocardiogram. White arrows point to two mobile left ventricular thrombi shown with white arrows. Akinetic distal anteroseptal wall and apex were also noted

Cardiac catheterization was prompted, which demonstrated critical stenosis of the proximal left anterior descending (LAD) artery and additional >80% stenosis of both the first diagonal artery and first obtuse marginal artery (Figure 3, Supplemental Figure 2). The right coronary artery was free of any significant obstructive disease.

**Figure 3.**
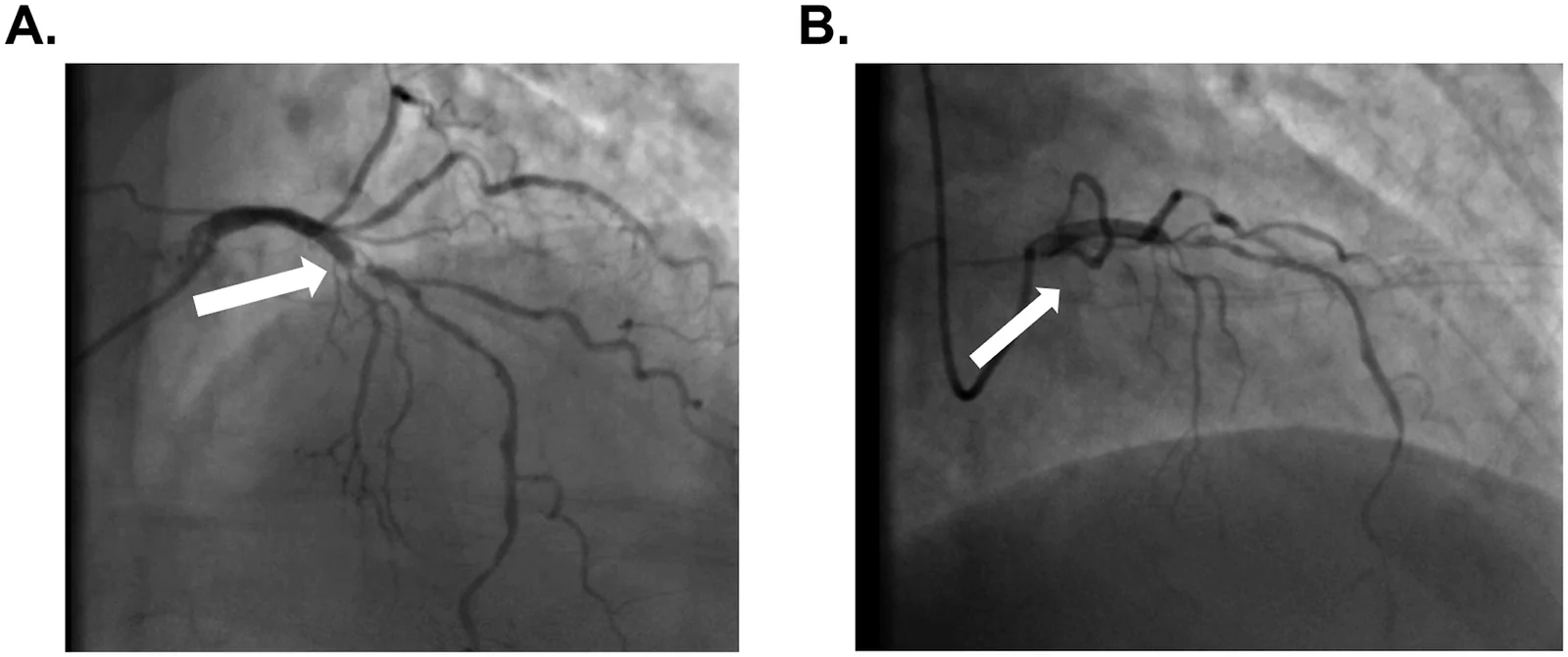
Cardiac catheterization. A) Stenosis in the diagonal coronary artery B) Stenosis in the proximal branches of the left anterior descending coronary artery as well as proximal diagonal disease

A multidisciplinary discussion weighing the therapeutic options was held. Notably, the patient’s rest pain and ischemic foot changes had progressed despite anticoagulation. In the presence of concomitant LV and popliteal thrombus, the decision was made to proceed with CABG, LV embolectomy, and popliteal artery thrombectomy in the same surgical setting, with the notion that surgery was more likely to achieve complete coronary and peripheral revascularization, thereby eliminating embolic risk.

In the operating room, cardiopulmonary bypass was performed simultaneously with left endoscopic vein harvesting. Longitudinal ventriculotomy lateral to the distal LAD was performed at the level of the LV apex (Figure 4A). Two pedunculated thrombi were directly identified and resected (Figure 4B). Subsequently, coronary revascularization was performed. Using the saphenous vein conduit harvested from the unaffected extremity, bypass was performed to the first obtuse marginal artery and first diagonal artery. The left internal mammary artery was grafted to the LAD, and the patient was rewarmed.

**Figure 4.**
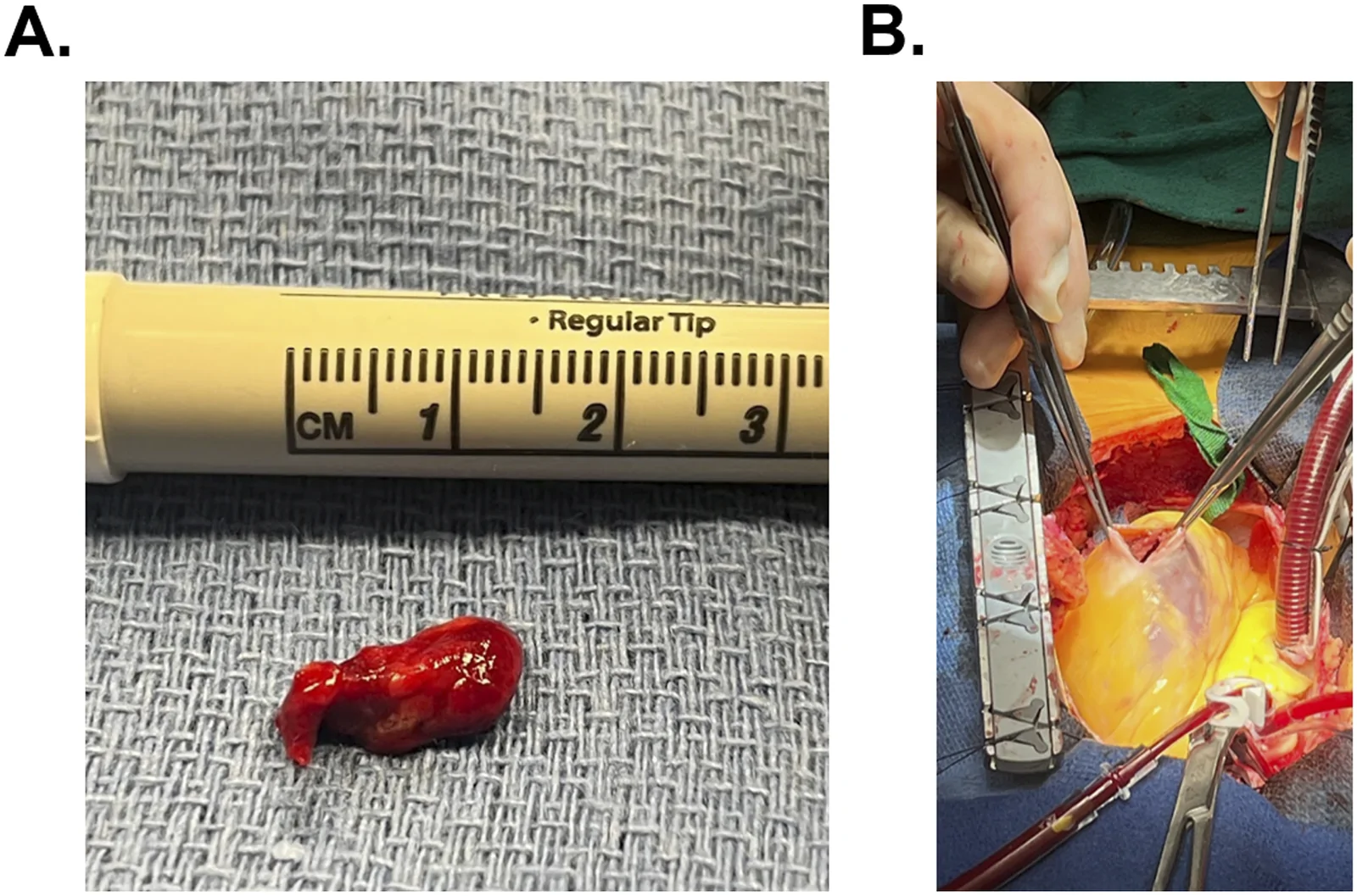
Intraoperative images. A) One of the LV thrombi that was excised following thrombectomy B) LV ventriculotomy performed through direct visualization lateral to the distal LAD at the level of the LV apex

Following the administration of heparin for cardiopulmonary bypass, right popliteal artery thrombectomy was performed. A segment of the saphenous vein was used to perform a patch angioplasty of the popliteal artery after obtaining adequate inflow and back bleeding. After the reversal of heparin, distal perfusion was assessed, and hemostasis was obtained in both wound beds.

The patient recovered in the intensive care unit and was extubated on the first postoperative day. She underwent hematologic workup, which was unremarkable, and was discharged to a rehabilitation facility due to deconditioning on postoperative day 6. She was maintained on dual antiplatelet therapy for three months postoperatively, along with lipid-lowering measures and beta blocker.

At three-month follow-up, the patient reported near complete resolution of symptoms. She participated in cardiac rehabilitation and denied any lower extremity claudication symptoms. Furthermore, hematology workup showed no evidence of hypercoagulability that could have potentially explained her LV thrombus. Her repeat echocardiogram showed no apical thrombus with near completely normal LV function. Long-term prognosis remains favorable, and she was continued on a single antiplatelet agent, LDL-lowering measures, and intense glycemic control to reduce cardiac risk factors. A timeline of the present case is demonstrated in Table 1.

**Table 1.** Timeline of the Case

Date	Events
Presentation	61-year-old woman presented to the emergency department with 10-day rest pain in right foot with progressive purple discoloration of toes
	Denies chest pain or shortness of breath
Day 1	Duplex ultrasonography demonstrated concern for popliteal artery thrombus
	Laboratory results: elevated troponin I (549 ng/L), WBC (11.9 k/μL), HbA1c (11.5%)
	Patient was systemically heparinized
	CT angiogram confirmed right popliteal artery occlusion
Day 2	TTE identified two highly mobile, pedunculated LV thrombi and akinetic anteroseptal wall with EF of 45-50%
	Cardiac catheterization revealed proximal LAD stenosis and >80% stenosis of diagonal and obtuse marginal arteries
Day 5	CABG (LIMA to LAD, SVG to diagonal and obtuse marginal), LV ventriculotomy with thrombus removal, and popliteal artery thrombectomy with patch angioplasty were performed in the same operative setting
POD 1	Patient was extubated in the ICU
POD 6	Following an unremarkable hospital course, patient was discharged to a rehabilitation facility for deconditioning
	Maintained on dual antiplatelet therapy and beta blocker
90-Day Follow-up	Clinically well with no sequelae
	Repeat echocardiogram demonstrated no apical thrombus with near completely normal LV function
	Denies any lower extremity claudication symptoms

WBC= white blood cell, HbA1c= Hemoglobin A1c, CT= computed tomography, TTE= transthoracic echocardiogram, LV= left ventricle, EF= ejection fraction, LAD= left anterior descending, CABG = coronary artery bypass grafting, LIMA= left internal mammary artery, SVG= saphenous vein graft, ICU= intensive care unit.

## Discussion

This report demonstrates multivessel CABG and concurrent LV and popliteal thrombectomy in a patient with limb malperfusion and advanced CAD. Despite advances in reperfusion therapy and novel anticoagulants, LV thrombus remains a significant complication in the setting of extensive anteroapical MI and multivessel CAD.^3^ LV thrombus can occur as frequently as 25% of acute MI cases and is associated with decreased myocardial salvage and more pronounced reperfusion injury.^1^ Further, LV thrombus generation after acute MI is associated with a 5.5-fold increase in embolic risk relative to patients without thrombus.^2^ Nearly 22% of these clots embolize systemically, often traveling to the common femoral arteries, pelvis, and cerebrum.^3^

Treatment of LV thrombus typically involves anticoagulation therapy and close follow-up with repeat imaging favored at 3 months following initial diagnosis. Previous guidelines have reported significant LV thrombus resolution following long-term treatment with traditional vitamin K-antagonists and direct oral anticoagulants.^8^ However, in cases when a patient is at high embolic risk, surgical removal may be warranted. In a recent review, Camaj and colleagues identified surgical management to be utilized often in the setting of another indication for cardiac surgery or at high-risk cardioembolic stroke.^1^ Potential risks of left ventriculotomy include further depressed LV function and arrhythmogenic potential postoperatively due to the fragility of recently infarcted myocardium. However, the mobile nature of LV thrombi warranted urgent surgical intervention in our patient due to the acute risk of embolization. Indeed, LV thrombus mobility and pedunculated morphology have been independently associated with greater odds of embolic potential, irrespective of size.^3,8^ Operative management in these patients is associated with reduced incidence of postoperative thromboembolism at follow-up relative to percutaneous or non-operative strategies.^9^ As such, our case aligns with contemporary literature prompting for surgical management of friable LV thrombi in patients with concurrent multivessel CAD.

Furthermore, the decision to proceed with CABG over high-risk percutaneous coronary intervention (PCI) in this case is supported by previous literature. Andre et al demonstrated CABG as a practical option with reduced risk of perioperative embolization in a patient with LV thrombus and severe CAD.^6^ A similar case by Gogayeva et al revealed a favorable outcome following urgent left ventriculotomy and multivessel CABG in a patient with a significant comorbid burden.^4^ Practice guidelines recommend surgical revascularization for patients with LV thrombus and severe CAD.^3^ However, a paucity of large-scale studies has compared PCI versus CABG in patients with friable LV thrombus. Given the history of embolization and the presence of multivessel CAD with associated LV thrombus, our group decided CABG would more likely achieve complete revascularization and eliminate embolic risk. Future studies evaluating optimal revascularization modality for these patients are warranted.

In addition, patients presenting with limb ischemia may require urgent revascularization to prevent irreversible tissue damage and limb loss. Contemporary management guidelines recommend prompt revascularization with prophylactic fasciotomy for a threatened yet salvageable limb.^7^ In the presence of multiple morbidities of acute MI, we report favorable outcomes following concurrent LV, popliteal thrombectomy, and multivessel CABG. Congruent with the present case, prior literature has demonstrated similar postoperative outcomes following concomitant versus staged CABG and peripheral vascular surgery.^10^ Our case adds to current literature demonstrating the safety of concurrent surgical approaches to treat patients with diseased coronary and peripheral limb vasculature, along with significant clot burden. Shared clinical decision-making between cardiac and vascular teams may expedite care for patients with systemic thrombotic burden and coronary artery stenosis. Urgent management of these complex patients may reduce stroke risk, microvascular obstruction, and irreversible limb tissue damage.

## Conclusions

Left ventricular thrombus formation is a recognized complication after acute MI, with a notable risk of stroke and peripheral embolization. Due to concurrent multivessel coronary disease, surgical removal of LV and popliteal thrombi was performed along with on-pump CABG. Collectively, our case highlights the effectiveness of prompt surgical revascularization in patients with multivessel CAD and elevated embolic risk. Future studies examining the postoperative outcomes of these operations performed simultaneously in institutional and statewide cohorts are warranted.

## Supplemental Material

Supplemental Material - Concomitant Left Ventricular and Popliteal Thrombectomy With Coronary Artery Bypass Grafting Supplemental Material for Concomitant Left Ventricular and Popliteal Thrombectomy With Coronary Artery Bypass Grafting by Jeffrey Balian, Dominick D’Antuono, John Conrad, Ali Gheissari, Vahram Ornekian in Journal of Investigative Medicine High Impact Case Reports
