# Supplementary material for: Concomitant Left Ventricular and Popliteal Thrombectomy With Coronary Artery Bypass Grafting
Source: J Investig Med High Impact Case Rep. 2026 Sep 16;14:23247096261491190. doi: 10.1177/23247096261491190 (PMC13724852; doi:10.1177/23247096261491190)
Supplement: Supplementary file 1 [file sj-zip-1-hic-10.1177_23247096261491190.zip › Supplemental Figure 1, 2 Caption.pdf]

Supplemental Figure 1: Video of four-chamber view of transthoracic echocardiogram The echocardiogram demonstrates apical hypokinesis with two mobile thrombi at the apicoseptal portion of the left ventricle. The midseptum and mid-lateral wall appear hyperkinetic

Supplemental Figure 2: Video of four-chamber view of cardiac catheterization The first obtuse marginal artery demonstrates severe proximal stenosis. The circumflex artery demonstrates proximal and mid-segment stenosis with diffuse disease. The LAD is a medium caliber vessel that wraps around the apex, with critical proximal stenosis at the level of the first diagonal artery and diffuse long-segment stenosis extending beyond the mid-vessel. The RCA demonstrates mild luminal irregularities. Slow flow is noted in all three coronary arteries, consistent with endothelial dysfunction.
